# Anti-Survival and Pro-Apoptotic Effects of 6-Shogaol on SW872 Human Liposarcoma Cells via Control of the Intrinsic Caspase Pathway, STAT-3, AMPK, and ER Stress

**DOI:** 10.3390/biom10101380

**Published:** 2020-09-28

**Authors:** Anil Kumar Yadav, Byeong-Churl Jang

**Affiliations:** Department of Molecular Medicine, College of Medicine, Keimyung University, 1095 Dalgubeoldaero, Dalseo-gu, Daegu 42601, Korea; aydaegu@gmail.com

**Keywords:** 6-shogaol, caspase-9, AMPK, STAT-3, ER stress, SW872

## Abstract

Notably, 6-Shogaol, a bioactive natural substance, has anticancer effects on many types of tumors. Up to date, the anticancer effect and mode of action of 6-Shogaol on liposarcoma are not known. In this study, we investigated whether 6-Shogaol inhibits the growth of SW872 and 93T449 cells, two different human liposarcoma cell lines. Of note, 6-Shogaol inhibited the growth of SW872 and 93T449 cells without affecting that of normal 3T3-L1 preadipocytes. Specifically, 6-Shogaol further induced the apoptosis of SW872 cells, as evidenced by nuclear DNA fragmentation, increased sub G1 population, activation of the intrinsic caspase pathway, and PARP cleavage. However, pretreatment with either z-VAD-fmk, a pan-caspase inhibitor, or N-acetylcysteine, an antioxidant, attenuated the 6-Shogaol’s growth-suppressive and apoptosis-inducing effects on SW872 cells. Moreover, 6-Shogaol activated AMPK while inhibited STAT-3 in SW872 cells, and siRNA-based genetic silencing of AMPK or STAT-3 considerably blocked the growth-suppressive and apoptotic response of 6-Shogaol to SW872 cells. Moreover, 6-Shogaol also upregulated the expression and phosphorylation of GRP-78, eIF-2α, ATF4, and CHOP, known ER stress markers, in SW872 cells, illustrating the induction of ER stress. These findings collectively demonstrate that 6-Shogaol has strong antigrowth and proapoptotic effects on SW872 cells through regulation of the intrinsic caspase pathway, oxidative stress, STAT-3, AMPK, and ER stress.

## 1. Introduction

Soft tissue sarcomas (STS) are rare solid malignant tumors with different histologies and are commonly characterized by aggressive characteristics locally and in distant metastases [1,2]. Liposarcoma (LS) represents one of the most common subtypes of STS and originates from adipocytes [3]. LS is associated with considerable morbidity and mortality and particularly poor prognosis, due to local recurrence and tendency to metastasize to lungs and liver [4,5]. Estimated 1.313 × 10^4^ new cases and 5350 deaths from soft tissue sarcoma have reported in the United States in 2020 [6]. Despite the development and clinical utilization of new targeted chemotherapeutic agents, improved radiation targeting, and surgical techniques, only minimal increases in sarcoma patients’ overall survival have been demonstrated in the last two decades [7]. There remains a great need for more effective and nontoxic antineoplastic drugs to enhance locoregional disease control as well as overall survival in sarcoma patients.

Apoptosis (programmed cell death) has received much attention as a possible mechanism for the elimination of extensively proliferating cancerous cells. It is accompanied by distinct morphological changes including plasma membrane blebbing, depolarization of mitochondria, chromatin condensation, and DNA fragmentation [8]. Several proteins are known to involve in apoptosis induction. Caspases are vital for the implementation of cell apoptosis triggered by different apoptotic stimuli [9]. Caspase activities are regulated by the family of the B-cell lymphoma-2 (Bcl-2) and inhibitor of apoptosis protein (IAP) [10,11]. Mounting of evidence has demonstrated the reduced expression of Bcl-2 and IAPs by anticancer agents in cancer cells, which is crucial for their anticancer mechanism [12,13]. AMP-activated protein kinase (AMPK) is a highly sensitive sensor of intracellular energy homeostasis [14]. Of note, a wealth of information illustrates that activation of AMPK contributes to the induction of apoptosis in several cancer cells exposed to anticancer drugs [15,16,17]. Signal transducer and activator of transcription-3 (STAT-3) belongs to the STAT family of proteins, which are both signal transducers and transcription factors [18]. In many solid tumors, STAT-3 is constitutively activated and provides neoplastic cells with proliferation signals with survival advantages, and genetic silencing or pharmacological inhibition of STAT-3 leads to the induction of growth suppression and apoptosis [19,20,21].

Increasing evidence also illustrates that the overload of the endoplasmic reticulum (ER) results in ER stress, which eventually leads to the inhibition of cell survival or the induction of cell death [22,23]. The ER is a highly dynamic organelle involved in various cellular functions such as protein synthesis, protein folding, trafficking, lipid synthesis, and calcium (Ca^2+^) regulation [24]. A number of chemotherapeutic drugs can trigger the accumulation of unfolded proteins, resulting in ER stress [25]. Severe or prolonged ER stress is characterized by not only overexpression of glucose-regulated protein (GRP-78), CCAAT enhancer-binding protein homologous protein (CHOP), and activating transcription factor (ATF-4) but also hyperphosphorylation of PKR-like endoplasmic reticulum kinase (PERK) and eukaryotic initiation factor 2-α (eIF2-α) [26,27,28]. Recent studies have also stressed a crucial role of oxidative stress through reactive oxygen species (ROS) production in the process of apoptosis induction in cancer cells [29].

Interestingly, 6-Shogaol is a bioactive ingredient in ginger (*Zingiber officinale* Roscoe) and has anticancer properties against many human cancer cells [30,31,32]. However, up to date, the anticancer effect and mechanism of action of 6-Shogaol in liposarcoma are unknown. In this study, we investigated whether 6-Shogaol inhibits the growth of SW872 (undifferentiated) and 93T449 (differentiated) cells, two different human liposarcoma cell lines. In this article, we report, for the first time, that 6-Shogaol has strong antigrowth effects on SW872 and 93T449 cells, and its growth-inhibitory and proapoptotic effects on SW872 cells are mediated through regulation of the intrinsic caspase pathway, oxidative stress, STAT-3, AMPK, and ER stress.

## 2. Materials and Methods

### 2.1. Chemicals and Antibodies

Briefly, 6-Shogaol (purity ≥ 99.84%) was purchased from Selleckchem (Houston, TX, USA). Dulbecco’s Modified Eagle’s Medium (DMEM) (LM-001-05), RPMI 1640 (LM-011-01), fetal bovine serum (FBS) (S001-01), and penicillin/streptomycin cocktail (LS202-02) were purchased from WelGENE Inc. (Daegu, Korea). Control siRNA (cat. no. sc-37007), AMPK siRNA (cat. no. sc-41102), and STAT-3 siRNA (cat. no. sc-29493) were purchased from Santa Cruz Biotechnology (Delaware, CA, USA). z-VAD-fmk was purchased from Calbiochem (Madison, WI, USA). Western Bright TM enhanced chemiluminescence (ECL, cat. no. K-12045-D20) was bought from Advansta Corporation (San Jose, CA, USA). Well plates (6 and 24 wells) and cell culture dishes (60 or 100 mm) were obtained from SPL Life Sciences (Gyeonggi-do, Korea). A detailed list of antibodies used in this study is included in Supplementary Table S1.

### 2.2. Cell Culture

Human SW872 (CRL-3043™), 93T449 (CRL-HTB-92™) liposarcoma cells, and mouse normal 3T3-L1 (CL-173™) preadipocyte cells (ATCC, Manassas, VA, USA) were grown in DMEM/RPMI-1640 with 10% heat-inactivated FBS (HI-FBS) and 1% penicillin/streptomycin at 37 °C in a humidified air (95% air and 5% CO_2_).

### 2.3. Cell Count and Morphological Analysis

SW872, 93T449, and 3T3-L1 cells were seeded in a 24-well plate with the density at 1 × 10^5^ cells/mL per well in the final volume of 500 μL. After overnight incubation, cells were treated with vehicle control (DMSO; 0.1%) or with 6-Shogaol or other drugs [z-VAD-fmk or N-acetyl-L-cysteine (NAC)] at the indicated concentrations for different periods (8 and 24 h). At each time point, the number of surviving cells, based on the principle that live cells have intact cell membranes, which cannot be stained with trypan blue dye (0.4%, cat. no. 15250-061, Gibco, Grand Island, NY, USA), were counted. Approximately 100 cells were counted for each evaluation. For cell morphology analysis, phase-contrast images of the conditioned cells treated with or without 6-Shogaol or transfected with siRNA (control, STAT-3, and AMPK) were taken with a phase-contrast microscope (Nikon Eclipse TS200, Nikon Corp., Tokyo, Japan).

### 2.4. Colony Formation Assay

SW872 cells were seeded at a density of 200 cells/well in 24-well plate. After overnight incubation, cells were treated with different concentrations of 6-Shogaol (1, 5, 10, and 20 μM) for two weeks. Colonies were fixed with 100% methanol and stained with 0.5% crystal violet [33].

### 2.5. Measurement of DNA Fragmentation

Evaluation of fragmented genomic DNA was processed as described previously [34]. Briefly, SW872 cells were seeded (1 × 10^5^ cells per mL) in 100-mm petri-plate the day before treatment. Cells were treated with vehicle control or 6-Shogaol and/or z-VAD-fmk for 24 h. Later, cells were collected, washed, and lysed in a buffer containing 50 mM Tris (pH 8.0), 0.5% sarkosyl, 0.5 mg/mL proteinase K, and 1 mM EDTA at 55 °C for 3 h, followed by the addition of RNase A (0.5 μg/mL) for a further 18 h at 55 °C. The lysates were then centrifuged at 1 × 10^4^× *g* for 20 min, genomic DNA in the supernatant was extracted with an equal volume of neutral phenol-chloroform-isoamyl alcohol mixture (25:24:1) and analyzed electrophoretically on a 1.8% agarose gel containing Gel Red nucleic acid stain (cat. no. 41003, Biotium, Fremont, CA, USA).

### 2.6. Quantification of Sub G1 Phase by Flow Cytometry Analysis

After 24 h treatment with vehicle control (DMSO; 0.1%), 6-Shogaol (20 µM) or other chemicals (z-VAD-fmk or NAC), SW872 cells were collected, washed with PBS, and fixed in ice-cold 70% ethanol at least for 2 h at 4 °C. Before the quantification, cells were washed once with PBS and incubated in 1 mL of cold propidium iodide solution comprising 100 μg/mL RNase A, 50 μg/mL propidium iodide, 0.1% (*w/v*) sodium citrate, and 0.1% (*v*/*v*) NP-40 and incubated on ice for 30 min in the darkness. Later, cytometric analyses were carried out using flow cytometer (FACS Caliber, Becton Dickinson, Franklin Lakes, NJ, USA) and CellQuest software (version 5.2, Becton Dickinson, Franklin Lakes, NJ, USA).

### 2.7. Preparation of Whole-Cell Lysate

SW872 cells (1 × 10^5^ cells/mL) were grown in 6-well plates. After overnight incubation, cells were treated with 6-Shogaol or other reagents, and at designated periods, SW872 cells were collected and lysed in RIPA buffer (Sigma-Aldrich; Merck; St. Louis, MO, USA) supplemented with proteinase inhibitor cocktail (1×). The cell lysates were centrifuged at 1.2074 × 10^4^× *g* for 20 min at 4 °C and supernatant was recovered, and protein concentration was determined using Bicinchoninic Acid (BCA) Protein Assay Kit (Thermo Scientific, Rockford, IL, USA).

### 2.8. Immunoblot Analysis

Proteins (50 µg) were loaded and run in 10% or 12% of SDS-polyacrylamide gel electrophoresis (SDS-PAGE). After separation of proteins, they were transferred onto polyvinylidene difluoride membrane (PVDF, Millipore, Bedford, MA, USA) and then blocked with 5% (*w*/*v*) skim milk in TBST for 1 h at room temperature (RT). Membranes were incubated with specific antibodies listed in Supplementary Table S1 at 4 °C. Later, membranes were rinsed with TBST buffer and further incubated with anti-goat IgG or anti-mouse IgG or anti-rabbit IgG coupled with horseradish peroxidase for 2 h at RT. Later, membranes were rinsed three times with TBST and developed with enhanced chemiluminescence (ECL) reagents. Actin expression levels were used as an equal protein loading control.

### 2.9. Small Interfering RNA (siRNA) Transfection

For small interfering RNA (siRNA) transfection, SW872 cells were seeded at a density of 1 × 10^5^ cells/mL into 6-well plates and transfected with a final concentration of 100 pM of control, AMPK, STAT-3 siRNA, using Lipofectamine^®^ RNAiMAX Transfection Reagent (Invitrogen, Waltham, MA, USA) for 6 h. Later, culture media of conditioned cells was replenished with fresh DMEM with 10% HI-FBS and incubated additionally for 18 h. After 24 h of transfection with AMPK siRNA, conditioned cells were treated with or without 6-Shogaol (20 µM) for the additional 24 h. The numbers of surviving cells were counted under the microscope and whole cell lysates were also prepared from the conditioned cells for Western blot analysis.

### 2.10. Measurement of Cellular ATP Contents

SW872 cells (0.3 × 10^5^ cells per well) were plated in 96-well plate and treated with or without 6-Shogaol (20 μM) or 2-deoxyglucose (2-DG), glucose mimetic that depletes levels of cellular ATP, for the indicated times and doses. Cellular ATP levels were assessed by ATPLite 1step (#6016941, PerkinElmer Inc., Waltham, MA, USA) according to the manufacturer’s protocol.

### 2.11. Statistical Analyses

Cell count analysis was performed in triplicate and repeated three times. Data were expressed as mean ± standard error. Statistical analysis was performed using SPSS 11.5 software (SPSS, Inc. Chicago, IL, USA). Data were subjected to one-way ANOVA, followed by Dunnett’s post hoc test. *p* < 0.05 was considered to indicate statistically significant differences.

## 3. Results

### 3.1. 6-Shogaol Strongly Reduces the Survival and Induces the Apoptosis of SW872 Human Liposarcoma Cells

**Figure 1:**
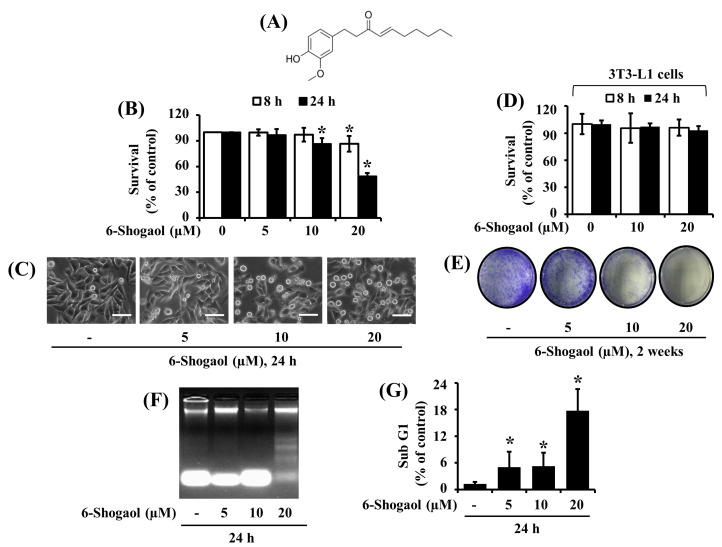
Effects of 6-Shogaol on the survival and apoptosis of SW872 liposarcoma cells. (**A**) The chemical structure of 6-Shogaol. (**B**) SW872 cells were treated with vehicle control (DMSO; 0.1%) or 6-Shogaol at the indicated concentrations and times. The survival rate was determined by cell counting assay. Experiments were performed in triplicate. Data are the means ± SE of three independent experiments. * *p* < 0.05 compared to the value of vehicle control at the indicated time. (**C**) SW872 cells were treated with vehicle control and 6-Shogaol at the indicated concentrations for 24 h. Images of the conditioned cells were obtained by phase-contrast microscopy, 400× (scale bar = 50 µm). Each image is representative of three independent experiments. (**D**) Normal 3T3-L1 preadipocyte cells were treated with vehicle control or 6-Shogaol at the indicated concentrations and times. The survival rate was determined by cell counting assay. The experiment was performed in triplicate. Data are the means ± SE of three independent experiments. (**E**) SW872 cells were treated with vehicle control or 6-Shogaol (20 µM) and incubated for 2 weeks, followed by crystal violet staining. Each image is representative of three independent experiments. (**F**) SW872 cells were treated with vehicle control or 6-Shogaol at the indicated concentrations for 24 h. Extranuclear fragmented DNA from the conditioned cells was extracted and analyzed on a 1.8% agarose gel. (**G**) After the abovementioned treatment in (**F**), the conditioned cells were harvested and subjected to fluorescence-activated cell sorting (FACS) analysis for measuring the population of sub G1 phase. Data are the means ± SE of three independent experiments. * *p* < 0.05 compared to the value of vehicle control at the indicated time.

Initially, we examined the effects of 6-Shogaol (Figure 1A) at different concentrations (5, 10, and 20 µM) and times (8 and 24 h) on the survival of SW872 human liposarcoma cells by cell count analysis. Treatment with 6-Shogaol at 20 μM most strongly reduced the survival of SW872 cells at 8 and 24 h (Figure 1B). Microscopic observations further revealed that 6-Shogaol caused a concentration-dependent decrease in the numbers of SW872 cells along with a dose-dependent increase in the numbers of round-shaped SW872 cells (Figure 1C). Similarly, 6-Shogaol treatment at the concentrations and times tested led to the reduced survival of 93T449 cells, another human liposarcoma cell line, along with the increased numbers of round-shaped cells (Figure S1A,B). Next, considering that liposarcoma cells originate from adipocytes [3], we measured the treatment effects of 6-Shogaol on the survival of normal 3T3-L1 preadipocytes. As shown in Figure 1D, 6-Shogaol treatment at 10 or 20 µM for 8 or 24 h did not reduce the normal cell survival. We next sought to explore whether 6-Shogaol inhibits the survival and proliferation of SW872 cells using a clonogenic assay. As shown in Figure 1E, compared with control cells (without 6-Shogaol), there was a markedly diminished colony formation of SW872 cells treated with 6-Shogaol at 10 or 20 μM for 2 weeks. Next, we investigated whether 6-Shogaol induces the apoptosis of SW872 cells using a DNA fragmentation experiment. As shown in Figure 1F, there was a strong induction of nuclear DNA fragmentation in SW872 cells treated with 6-Shogaol at 20 µM for 24 h. Data of flow cytometry analyses, as shown in Figure 1G, also revealed that 6-Shogaol treatment at 20 µM led to the highest accumulation of sub G1 phase of SW872 cells. Since SW872 cells are more aggressive and tumorigenic than 93T449 cells [35], and the 20 μM concentration of 6-Shogaol caused the strongest antisurvival and proapoptotic effects, we selected SW872 cells and this 20 μM concentration of 6-Shogaol for further studies.

### 3.2. 6-Shogaol Induces the Intrinsic Caspase Activation-Dependent Apoptosis of SW872 Human Liposarcoma Cells

**Figure 2:**
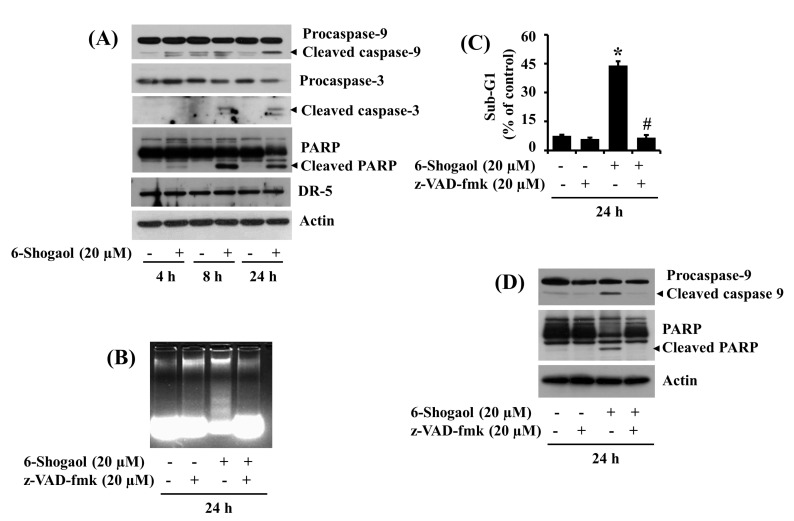
Effects of 6-Shogaol and/or z-VAD-fmk on the expression of procaspase-9/3, PARP, DR-5, nuclear DNA fragmentation, and sub G1 phase in SW872 cells. (**A**) SW872 cells were treated with or without 6-Shogaol (20 μM) for the designated periods. At each time point, whole cell lysates were prepared and analyzed by Western blotting with respective antibodies. (**B**) SW872 cells were treated with or without 6-Shogaol (20 μM) in the absence or presence of the pan-caspase inhibitor z-VAD-fmk (20 μM) for 24 h. Extranuclear fragmented DNA was extracted and analyzed on a 1.8% agarose gel. (**C**) After the treatment mentioned above in (**B**), the conditioned cells were harvested and subjected to fluorescence-activated cell sorting (FACS) analysis for measuring the cell population of the sub G1 phase. Data are the means ± SE of three independent experiments. * *p* < 0.05 compared to the vehicle control; # *p* < 0.05 compared with 6-Shogaol-treatment. (**D**) After the treatment mentioned above in (**B**), whole cell lysates from the conditioned cells were prepared and analyzed by Western blotting using respective antibodies.

**Figure 3:**
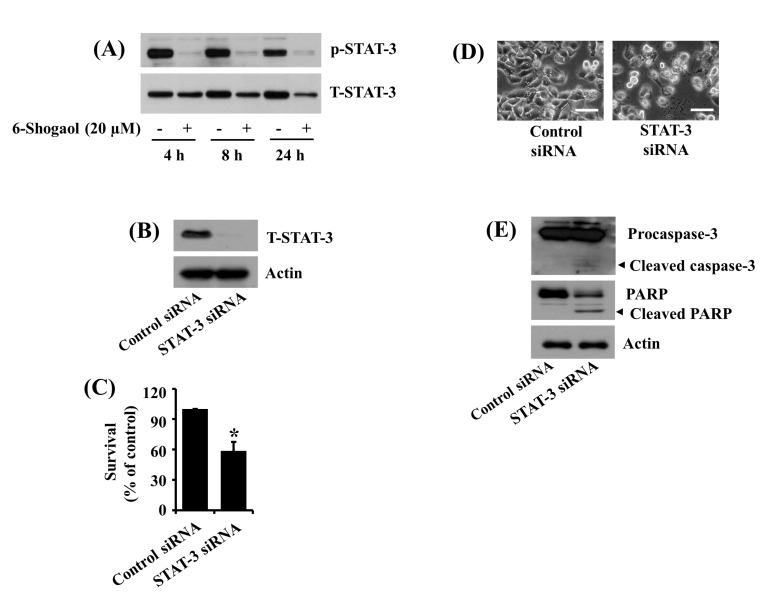
Effects of 6-Shogaol or gene silencing of STAT-3 on the expression and/or phosphorylation levels of STAT-3, procaspase-3, PARP cleavage, and survival of SW872 cells. (**A**) SW872 cells were treated with vehicle control (DMSO; 0.1%) or 6-Shogaol (20 µM) for the designated periods. At each time point, whole cell lysates from the conditioned cells were prepared and analyzed by Western blotting with respective antibodies. p-STAT-3, phosphorylated STAT-3; T-STAT-3, total STAT-3. (**B**) SW872 cells were transfected with 100 pM of control or STAT-3 siRNA for 24 h. Whole cell lysates were prepared and analyzed by Western blotting with respective antibodies. (**C**) After the treatment mentioned above in (**B**), the survival rate was determined by cell counting assay. The experiment was performed in triplicate. Data are the means ± SE of three independent experiments. * *p* < 0.05 compared to the control at the indicated time. (**D**) Images of control or STAT-3 siRNA-transfected cells in the same as in (**B**) were obtained by phase-contrast microscopy, 400× (scale bar = 50 µm). (**E**) After the treatment mentioned above in (**B**), whole cell lysates from the conditioned cells were prepared and analyzed by Western blotting with respective antibodies.

Next, using Western blotting, we studied the treatment effects of 6-Shogaol at 20 µM for 24 h on the expression levels of caspases, death receptor (DR), and PARP in SW872 cells over time. As shown in Figure 2A, 6-Shogaol treatment led to a time-dependent increase in the expression levels of active (proteolytically cleaved) caspase-9/3 and of cleaved PARP, a downstream effector of these caspases, in SW872 cells. In contrast, there was no alteration of the expression levels of DR-5 in SW872 cells treated with 6-Shogaol for the times tested. The expression levels of control actin protein remained constant under these experimental conditions. Next, we questioned whether the activation of caspases is crucial for the 6-Shogaol-induced apoptosis in SW872 cells using z-VAD-fmk, a pan-caspase inhibitor. Profoundly, z-VAD-fmk treatment at 20 µM greatly abrogated the 6-Shogaol-induced apoptotic parameters, including nuclear DNA fragmentation (Figure 2B) and accumulation of sub G1 phase cells (Figure 2C), in SW872 cells. Moreover, this pan-caspase inhibitor attenuated the 6-Shogaol-induced activation of caspase-9 and generation of cleaved PARP in SW872 cells (Figure 3D). The expression levels of control actin protein remained unchanged under these experimental conditions.

### 3.3. 6-Shogaol Inhibits STAT-3 Phosphorylation in SW872 Human Liposarcoma Cells, and STAT-3 Knockdown Causes Caspase-3 Activation, PARP Cleavage, and Reduction of SW872 Cell Survival

Several studies have demonstrated that STAT-3 is aberrantly activated and plays a crucial role in tumor initiation and progression in many cancers, including STS [19,20,21]. This promptly led us to investigate whether STAT-3 is expressed and phosphorylated in SW872 cells, and 6-Shogaol modulates these. Notably, in the absence of 6-Shogaol, there were high expression and phosphorylation levels of STAT-3 in SW872 cells at times tested (Figure 3A). However, treatment with 6-Shogaol strongly diminished the phosphorylation levels of STAT-3 at times applied. Of further interest, although 6-Shogaol treatment at 4 h did not affect the total protein expression levels of STAT-3, 6-Shogaol treatment at 8 or 24 h resulted in a considerable decrease in the protein’s total expression levels. We further confirmed the role of STAT-3 inhibition in the 6-Shogaol-induced reduction of SW872 cell survival using STAT-3 siRNA transfection. As clearly shown in Figure 3B, there was a complete loss of endogenous STAT-3 expression in STAT-3 siRNA-transfected SW872 cells compared with the control siRNA-transfected ones. Subsequent cell count analysis and microscopic observation data showed considerable growth inhibition of STAT-3 siRNA-transfected SW872 cells compared with control siRNA-transfected ones (Figure 3C,D). Of further importance, the STAT-3 knockdown led to the activation of caspase-3 and PARP cleavage in SW872 cells (Figure 3E). The expression levels of control actin protein remained constant under these experimental conditions.

### 3.4. 6-Shogaol Induces AMPK Activation and ATP Depletion in SW872 Human Liposarcoma Cells, and AMPK Knockdown Mitigates the 6-Shogaol-Induced Reduction of SW872 Cell Survival

**Figure 4:**
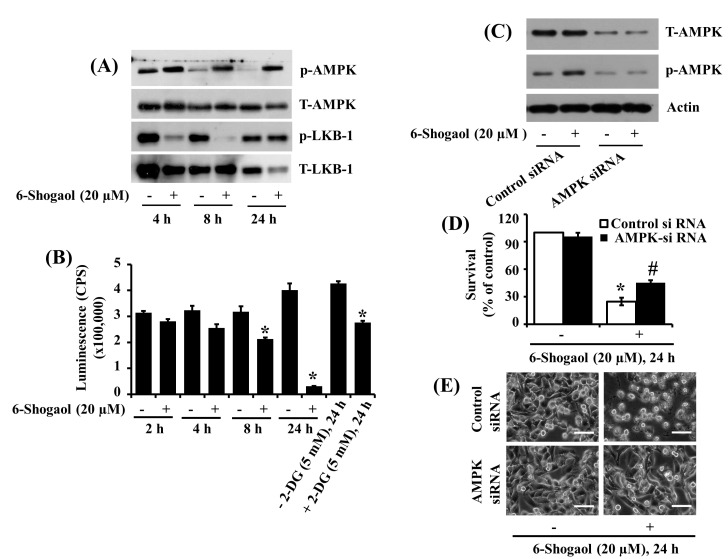
Effects of 6-Shogaol or gene silencing of AMPK on the expression and/or phosphorylation of AMPK, LKB-1, cellular ATP content, and survival of SW872 cells. (**A**) SW872 cells were treated with vehicle control (DMSO; 0.1%) or 6-Shogaol (20 µM) for the designated periods. At each time point, whole cell lysates were prepared and analyzed by Western blotting with respective antibodies. p-AMPK, phosphorylated AMPK; T-AMPK, total AMPK; p-LKB-1, phosphorylated LKB-1; T-LKB-1, total LKB-1. (**B**) SW872 cells were treated with vehicle control or 6-Shogaol (20 µM) for the designated periods. Deoxyglucose (2-DG), a known ATP depleting agent, was included as positive control. At each time point, cellular ATP content was measured. * *p* < 0.05 compared to the value of 6-Shogaol or 2-DG free control at the indicated time. (**C**) SW872 cells were transfected with 100 pM of control or AMPK siRNA for 24 h. Control- or AMPK siRNA-transfected cells were treated with vehicle control or 6-Shogaol (20 μM) for 24 h. Whole cell lysates were prepared and analyzed by Western blotting with respective antibodies. (**D**) After the treatment mentioned above in (**C**), the survival rate was determined by cell counting assay. The experiment was performed in triplicate. Data are the means ± SE three independent experiments. * *p* < 0.05 compared to the control at the indicated time; # *p* < 0.05 compared to 6-Shogaol treated at the indicated time. (**E**) After the treatment mentioned above in (**C**), images of the conditioned cells were obtained by phase-contrast microscopy, 400× (scale bar = 50 µm).

Next, we investigated the treatment effects of 6-Shogaol at 20 µM on the expression and phosphorylation (T172) of AMPK and liver kinase B-1 (LKB-1), an upstream activator of AMPK [36], in SW872 cells over time. Of note, as shown in Figure 4A, in the absence of 6-Shogaol, there was substantial phosphorylation of AMPK in SW872 cells at 4 h, followed by a drastic decline of the protein phosphorylation levels thereafter. Strikingly, 6-Shogaol treatment resulted in robust and sustained phosphorylation (activation) of AMPK in SW872 cells, particularly at 4 and 8 h. In the case of LKB-1, there was a gradual decline of its phosphorylation levels in control SW872 cells at times tested. However, 6-Shogaol treatment at 4 or 8 h led to a large reduction of LKB-1 phosphorylation levels in SW872 cells. There was no difference in LKB-1 phosphorylation levels in SW872 cells treated without or with 6-Shogaol for 24 h. The total protein expression levels of AMPK and LKB-1 remained constant under these experimental conditions of 4 and 8 h, but their levels weakly and strongly decreased at 24 h. Given that AMPK activation is controlled by the change of cellular AMP/ATP ratio [37], we next determined whether 6-Shogaol lowers cellular ATP content in SW872 cells. For comparison, 2-deoxyglucose (2-DG) that lowers cellular ATP content [38] was included as a positive control. Moreover, 6-Shogaol treatment decreased cellular ATP content in the SW872 cells in a time-dependent manner (Figure 4B). Apparently, in SW872 cells, the reduced levels of cellular ATP content induced by 6-Shogaol treatment with 20 μM for 8 and 24 h were higher than those by 2-DG treatment with 5 mM for 24 h. Using AMPK siRNA transfection, we next asked whether AMPK activation plays an important role in the 6-Shogaol-induced reduction of SW872 cell survival. As shown in Figure 4C, there was much lower protein expression and phosphorylation levels of AMPK in AMPK siRNA-transfected SW872 cells compared with control siRNA-transfected ones, illustrating the AMPK siRNA transfection efficiency. The expression levels of control actin protein remained unchanged under these experimental conditions. Importantly, data of cell count analysis demonstrated a much higher survival of the AMPK siRNA-transfected SW872 cells than control siRNA-transfected cells (Figure 4D). Microscopic observations, as shown in Figure 4E, further illustrated that although 6-Shogaol treatment at 24 h caused a marked increase in the numbers of round-shaped SW872 cells transfected with control siRNA, there was much less number of round-shaped cells transfected with AMPK siRNA.

### 3.5. NAC Attenuates the 6-Shogaol-Induced Reduction of Survival and Apoptosis of SW872 Human Liposarcoma Cells

**Figure 5:**
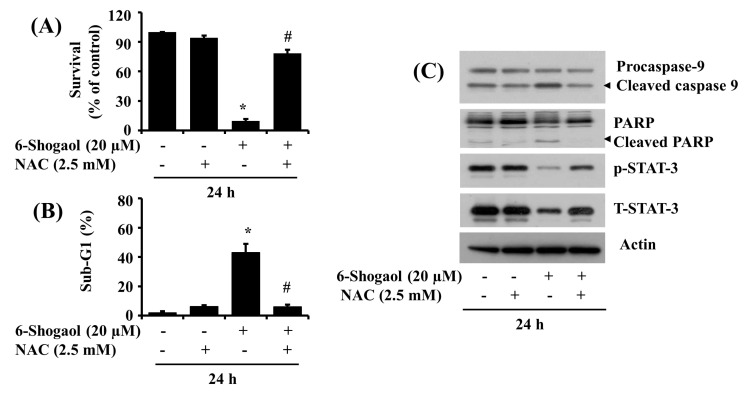
Effects of 6-Shogaol and/or NAC on the survival, sub G1 phase, and expression and phosphorylation of procaspase-9, PARP, and STAT-3 in SW872 cells. (**A**) SW872 cells were treated with or without 6-Shogaol (20 μM) in the absence or presence of NAC (2.5 mM) for 24 h, followed the measurement of survival rate by cell counting assay. The experiment was performed in triplicate. Data are the means ± SE of three independent experiments. * *p* < 0.05 compared to the vehicle control; # *p* < 0.05 compared with 6-Shogaol treated. (**B**) After the treatment mentioned above in (**A**), the conditioned cells were harvested and subjected to fluorescence-activated cell sorting (FACS) analysis for measuring the cell population of the sub G1 phase. Data are the means ± SE of three independent experiments. * *p* < 0.05 compared to the vehicle control; # *p* < 0.05 compared with 6-Shogaol treated. (**C**) After the treatment mentioned above in (**A**), whole cell lysates were prepared and analyzed by Western blotting with respective antibodies. PARP, poly (ADP-ribose) polymerase; p-STAT-3, phosphorylated STAT-3; T-STAT-3, total STAT-3.

Many anticancer drugs eliminate tumor cells by inducing apoptosis through oxidative stress via generation of ROS [29]. Increasing evidence also illustrates the role of ROS production in the 6-Shogaol-induced apoptosis of certain cancer cells [32,39]. We thus questioned whether the 6-Shogaol-induced growth inhibition and apoptosis of SW872 cells seen herein are due to ROS production using NAC, an antioxidant [40]. Notably, NAC treatment at 2.5 mM strongly reversed not only the 6-Shogaol-induced reduction of SW872 cell survival (Figure 5A) but also the 6-Shogaol-induced accumulation of sub G1 phase cells (Figure 5B). Moreover, this antioxidant effectively blocked not only the 6-Shogaol-induced activation of caspase-9 and PARP cleavage but also the 6-Shogaol-induced downregulation of STAT-3 phosphorylation and expression in SW872 cells (Figure 5C). The expression levels of control actin protein remained constant under these experimental conditions.

### 3.6. 6-Shogaol Induces the Altered Expression and Phosphorylation Levels of GRP-78, eIF-2α, ATF-4, CHOP, mTOR, 4EBP-1, and S6 in SW872 Human Liposarcoma Cells

**Figure 6:**
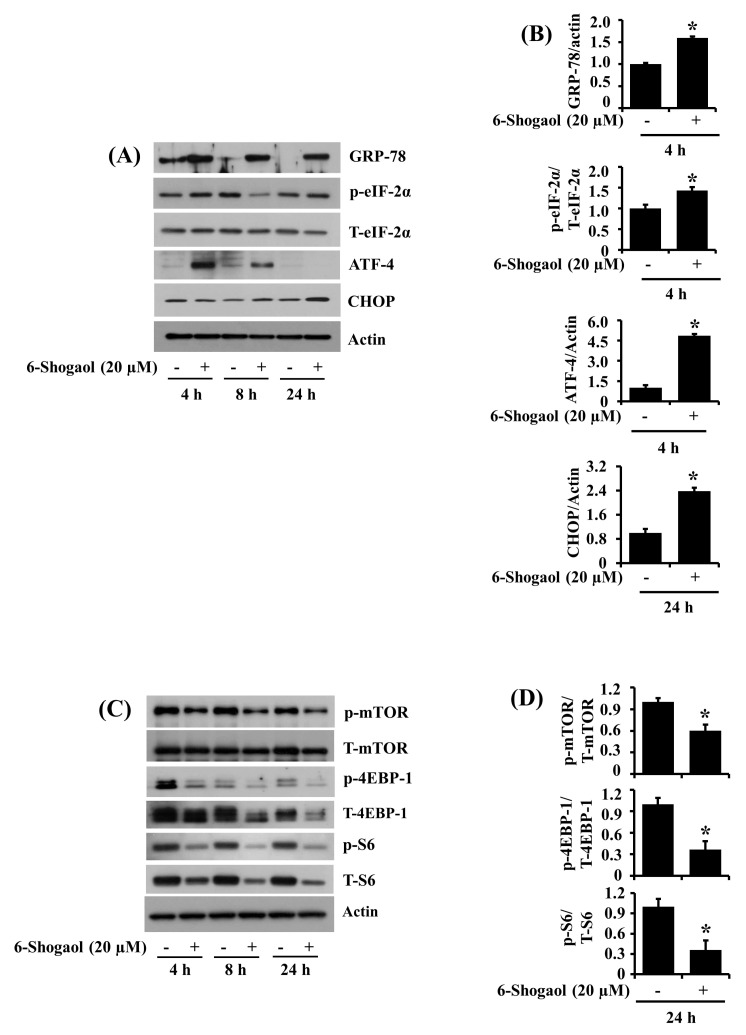
Effects of 6-Shogaol on the expression and phosphorylation levels of GRP-78, eIF-2α, ATF-4, CHOP, mTOR, 4EBP-1, and S6 in SW872 cells. (**A**) SW872 cells were treated with vehicle control (DMSO; 0.1%) or 6-Shogaol (20 µM) for the designated periods. At each time point, whole cell lysates were prepared and analyzed by Western blotting with respective antibodies. p-eIF-2α, phosphorylated eIF-2α; T-eIF-2α, total eIF-2α. (**B**) The densitometry data of Figure S2A,B. * *p* < 0.05 compared to the control at the indicated time. (**C**) SW872 cells were treated with vehicle control or 6-Shogaol (20 µM) for the designated periods. At each time point, whole cell lysates were prepared and analyzed by Western blotting with respective antibodies. p-mTOR, phosphorylated mTOR; T-mTOR, total mTOR; p-4EBP-1, phosphorylated 4EBP-1; T-4EBP-1, total 4EBP-1; p-S6, phosphorylated S6; T-S6, total S6. (**D**) The densitometry data of Figure S2C. * *p* < 0.05 compared to the vehicle control at the indicated time.

Given that severe or prolonged ER stress and the resultant global translation blockage leads to inhibition of cancer cell survival and/or induction of cancer cell death [22], we next sought to explore whether 6-Shogaol modulates the expression and phosphorylation levels of ER stress and translation-related proteins in SW872 cells. As shown in Figure 6A, in the absence of 6-Shogaol, there was a time-dependent decrease in the protein expression levels of GRP-78 in SW872 cells. However, the 6-Shogaol treatment led to a robust and sustained increase in the protein expression levels of GRP-78 in SW872 cells at times tested. Furthermore, 6-Shogaol treatment at 4 and 24 h resulted in a slight increase in the phosphorylation levels of eIF-2α in SW872 cells without affecting its total protein levels. Moreover, 6-Shogaol treatment at 4 or 8 h markedly upregulated expression of ATF-4 in SW872 cells. Additionally, 6-Shogaol treatment at 8 and 24 h resulted in a slight or strong induction in the expression levels of CHOP in SW872 cells. The densitometry data of the triplicate experiment also confirmed the ability of 6-Shogaol treatment at 4 or 24 h to increase the expression and phosphorylation levels of GRP-78, eIF-2α, CHOP, and ATF-4 in SW872 cells (Figure 6B and Figure S2A,B). As shown in Figure 6C, in the absence of 6-Shogaol, there was a substantial expression and phosphorylation levels of mTOR, 4EBP-1, and S6 in SW872 cells at times tested. Strikingly, the 6-Shogaol treatment caused a time-dependent decrease in both expression and phosphorylation levels of these proteins in SW872 cells. The densitometry data of the triplicate experiment further revealed the ability of 6-Shogaol treatment at 24 h to downregulate the expression and phosphorylation levels of mTOR, 4EBP-1, and S6 in SW872 cells (Figure 6D and Figure S2C).

## 4. Discussion

Up to date, the anti-cancer effect and mode of action of 6-Shogaol, a bioactive constituent in ginger, in human liposarcoma are unknown. In this study, we have demonstrated that 6-Shogaol has anti-survival and pro-apoptotic effects on SW872 human liposarcoma cells, and these effects are mediated through regulation of the intrinsic caspase pathway, oxidative stress, STAT-3, AMPK, and ER stress.

Studies have previously shown that 6-Shogaol has anti-proliferative, anti-survival, and pro-apoptotic effects on many different types of human cancer cells, such as SMMC-7721 (liver), HGC (gastric), A2780 (ovarian), COLO 205 (colon), A549 (lung), HeLa (cervix), and MDA-MB-231 (breast) [30,31,32,39,41,42,43]. The known molecular mechanisms underlying 6-Shogaol’s anticancer effects include the induction of apoptosis, the activation of caspase-9, -8, -3, the accumulation of sub G1 phase cells [30,42,43,44,45,46], and the arrest of the cell cycle [47]. Through initial experiments, we herein have demonstrated that 6-Shogaol at 20 μM largely reduces the survival of SW872 and 93T449 human liposarcoma cells, but it does not affect normal 3T3-L1 preadipocytes, suggesting the specificity of 6-Shogaol to inhibit adipocytic tumor cells. The present study has further shown that 6-Shogaol at 20 μM strongly induces the apoptosis of SW872 cells, as judged by its abilities to induce the nuclear DNA fragmentation, the accumulation of sub G1 phase cells, the activation of caspase-9/3, and the cleavage of PARP. It is documented that apoptosis induction is mainly mediated through the intrinsic (mitochondrial) and extrinsic (DR)-mediated pathways in which either the mitochondria-mediated activation of caspase-9 or the DR-dependent activation of caspase-8 mediates events, respectively [8]. Thus the present findings that 6-Shogaol at 20 μM leads to the activation of caspase-9/3, but it does not influence the expression levels of DR-5 in SW872 cells and z-VAD-fmk, a pan-caspase inhibitor, strongly abrogates the ability of 6-Shogaol to induce apoptosis of SW872 cells highlight that 6-Shogaol selectively activates the intrinsic caspase pathway in SW872 cells, which is crucial for the 6-Shogaol-induced apoptosis in these cells.

The family of STATs plays an essential role in cancer cell survival, proliferation, and apoptosis [48,49]. Among the STAT family, STAT-3 has been extensively studied due to its constitutive expression in many human cancers, including soft tissue sarcoma cells [18,19,20,21]. Crucially, a wealth of information further illustrates that STAT-3 activation contributes to tumor cell survival, proliferation, invasion, and metastasis [50], and STAT-3 inhibition leads to suppression of the growth of numerous cancers in vitro and in vivo [19,20,21,51,52]. Notably, there are previous studies illustrating that 6-Shogaol suppresses the phosphorylation of STAT-3, which partially mediates its antiproliferative and proapoptotic effects on certain cancer cells [32,44,53]. However, until now, 6-Shogaol regulation of STAT-3 expression and phosphorylation levels in human liposarcoma cells is unknown. Notably, in this study, we have found that the short-term (4 h) treatment with 6-Shogaol at 20 µM greatly reduces the phosphorylation levels of STAT-3 without affecting the protein’s total expression levels, whereas the long-term (8 or 24 h) treatment substantially decreases both the phosphorylation and expression levels of STAT-3 in SW872 cells. The former may be due to the 6-Shogaol’s ability to directly or indirectly inhibit the phosphorylation of STAT-3 from the pre-existed STAT-3 proteins in SW872 cells, whereas the latter may be associated with the ability of 6-Shogaol to induce STAT-3 translational repression or protein turnover (disability) in these cells. It is of further importance demonstrated herein that knockdown of endogenous STAT-3 leads to a considerable growth inhibition and the induction of apoptosis, as evidenced by the activation of caspase-9/3 and the cleavage of PARP in SW872 cells. These results strongly suggest that STAT-3 is crucial for the survival of SW872 cells and STAT-3 inhibition crucially contributes to the 6-Shogaol-induced growth-suppressive and proapoptotic effects on SW872 cells.

AMPK is a master regulator of energy homeostasis and also controls in cell growth, autophagy, metabolism, and protein synthesis [14,54]. Accordingly, AMPK activation (phosphorylation on T172) leads to the growth inhibition and apoptosis induction in different cancer cells exposed to anticancer drugs [15,16,17]. Supporting this, we also have recently demonstrated that AMPK is highly expressed and phosphorylated in 93T449 human liposarcoma cells, and knockdown of endogenous AMPK leads to a considerable reduction of 93T449 cell survival [21], highlighting that AMPK acts as a survival factor in these cells. Interestingly, a previous study has reported that 6-Shogaol at 50 μM for 3 to 24 h inhibits AMPK in HepG2 human liver cancer cells [55]. Given that 6-Shogaol regulation of AMPK in liposarcoma cells is unknown, we herein have investigated whether AMPK is also expressed and phosphorylated in SW872 cells, and 6-Shogaol at 20 µM modulates it. Profoundly, we have found that 6-Shogaol at 20 µM induces a robust and sustained AMPK phosphorylation in SW872 cells, and gene silencing of AMPK partially attenuates the ability of 6-Shogaol to reduce SW872 cell survival. These results point out that AMPK also acts as a survival factor in SW872 cells, and AMPK inhibition may further contribute to the 6-Shogaol-induced anti-survival effects on these cells. Evidence suggests that AMPK phosphorylation (activation) is controlled by the activity of upstream kinases, such as LKB-1 [36], and the change of cellular ATP content [37]. Thus, assuming our present findings that 6-Shogaol at 20 µM greatly elevates AMPK phosphorylation, inhibits LKB-1 phosphorylation, and lowers cellular ATP content in SW872 cells, it is likely that AMPK activation by 6-Shogaol in SW872 cells is not through the LKB-1 pathway but through a reduced cellular ATP content.

Multiple lines of evidence strongly indicate that the induction of oxidative stress and ER stress mediates the growth-suppressive and apoptosis-inducing effects of 6-Shogaol on many types of human cancer cells [30,42,55]. Up to date, there is a lack of information about 6-Shogaol regulation of oxidative stress and ER stress in liposarcoma cells. Oxidative stress, a common mediator of apoptosis [56], occurs due to the imbalance of cellular oxidation and reduction status [57]. In this study, NAC, an antioxidant, greatly attenuates not only the 6-Shogaol-induced anti-survival and proapoptotic effects on SW872 cells but also the 6-Shogaol-induced activation of caspase-9 and PARP cleavage in these cells. Furthermore, NAC largely interferes with the ability of 6-Shogaol to downregulate the expression and phosphorylation levels of STAT-3 in SW872 cells. These results illustrate that oxidative stress is also crucial to the 6-Shogaol-induced anti-survival and proapoptotic effects on SW872 cells, and oxidative stress lies upstream of STAT-3 expression and activation in these cells. It is documented that cells undergoing severe or prolonged ER stress have distinct characteristics, including the abnormal accumulation of unfolded and misfolded (nonfunctional) proteins, the overexpression of molecular chaperones (e.g., GRP-78) and transcription factors (e.g., ATF-4), the hyperphosphorylation of eIF-2α, and the inhibition of global translation [58]. It is worth mentioning that eIF-2α is phosphorylated in response to cellular stress, and its phosphorylation leads to reduced protein synthesis [25]. Accordingly, GRP-78 is a master regulator of the unfolded protein response [26]. ATF-4 is a transcription factor that regulates a broad range of genes, which play an essential role in recovery from ER stress [59]. It has been well documented that transcription factor CCAAT enhancer-binding protein homologous protein (CHOP) is involved in ER stress-induced apoptosis [60]. Thus, considering that 6-Shogaol at 20 µM upregulates not only the expression of GRP-78, ATF4, and CHOP but also the phosphorylation of eIF-2α in SW872 cells herein, it is suggested that 6-Shogaol induces ER stress in SW872 cells. mTOR, p70S6K, S6, and 4EBP-1 are critical proteins involved in translation [61,62]. Accordingly, mTOR regulates protein synthesis by hyperphosphorylation of 4EBP-1, decreasing its affinity for the translation initiation factor eIF4E [63]. mTOR also phosphorylates and activates p70S6K, which in turn phosphorylates the 40S ribosomal subunit S6 protein [64]. In this study, 6-Shogaol at 20 µM substantially reduces the expression and phosphorylation levels of mTOR, 4EBP-1, and S6 in SW872 cells. Given that 6-Shogaol inhibits eIF-2α, mTOR, 4EBP-1, and S6, translation-related proteins, in SW872 cells herein, the 6-Shogaol’s anti-survival and proapoptotic effects are further likely to be mediated through the inhibition of global translation.

In summary, this is the first study reporting that 6-Shogaol has strong anti-survival and proapoptotic effects on SW872 human liposarcoma cells, and these effects are mediated through regulation of the intrinsic caspase pathway, oxidative stress, STAT-3, AMPK, and ER stress. Although there are important issues that need to be resolved, including the antitumor effect of 6-Shogaol on animal models, our present findings suggest that 6-Shogaol may be used as a potential therapeutic agent for the treatment of human liposarcoma.

## Supplementary Materials

The following are available online at https://www.mdpi.com/2218-273X/10/10/1380/s1. Figure S1. Effects of 6-Shogaol on the survival of 93T449 cells. Figure S2. Effects of 6-Shogaol on the expression and phosphorylation levels of GRP-78, eIF-2α, ATF-4, CHOP, mTOR, 4EBP-1, and S6 in SW872 cells. Table S1: List of antibodies used for Western blot analysis.
