# Supplementary material for: Anti-Survival and Pro-Apoptotic Effects of 6-Shogaol on SW872 Human Liposarcoma Cells via Control of the Intrinsic Caspase Pathway, STAT-3, AMPK, and ER Stress
Source: Biomolecules. 2020 Sep 28;10(10):1380. doi: 10.3390/biom10101380 (PMC7650770; doi:10.3390/biom10101380)
Supplement: Supplementary file 1 [file biomolecules-10-01380-s001.zip › biomolecules-936149-supplementary/Figure S1 and S2.pdf]

Figure S1

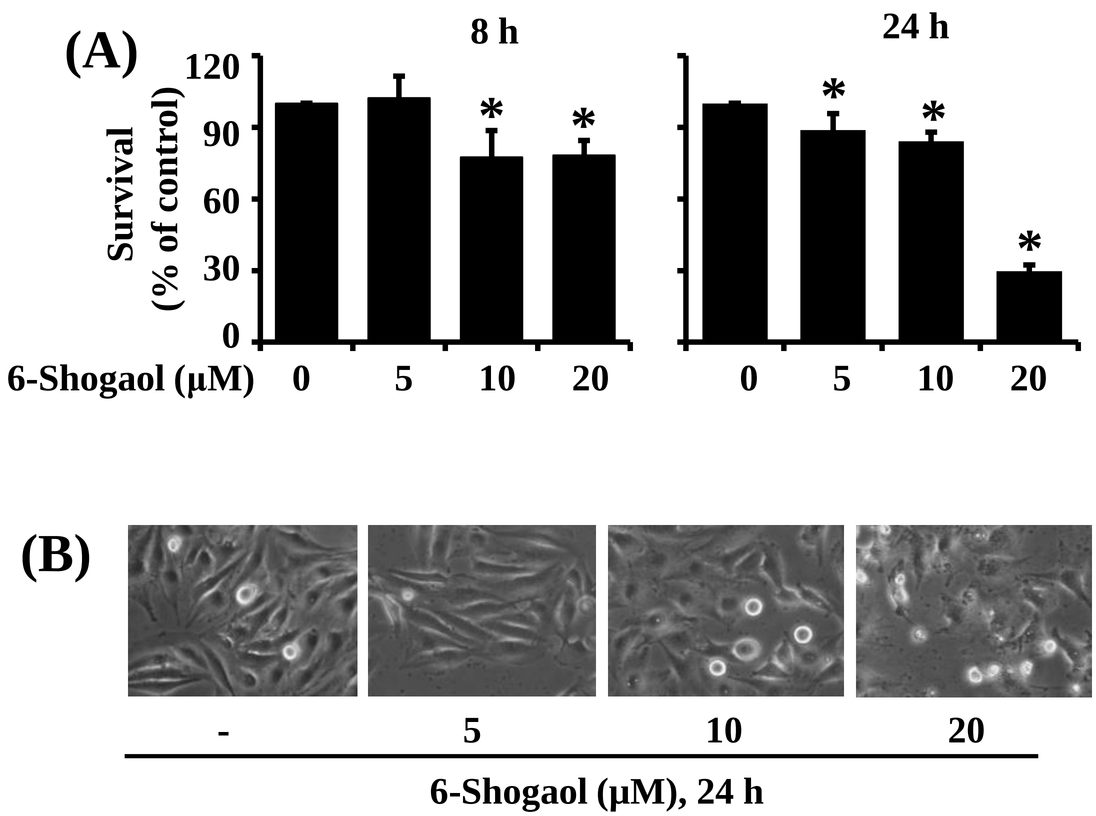

Figure S1. Effects of 6-Shogaol on the survival of 93T449 cells. **(A)** 93T449 cells were treated with vehicle control (DMSO; 0.1%) or 6-Shogaol at the indicated concentrations and times. The survival rate was determined by cell counting assay. Experiments were performed in triplicate. Data are the means ± SE of three independent experiments. * $p < 0.05$ compared to the value of vehicle control at the indicated time. **(B)** 93T449 cells were treated with vehicle control and 6-Shogaol at the indicated concentrations for 24 h. Images of the conditioned cells were obtained by phase-contrast microscopy, 400 ×. Each image is representative of three independent experiments.

Figure S2

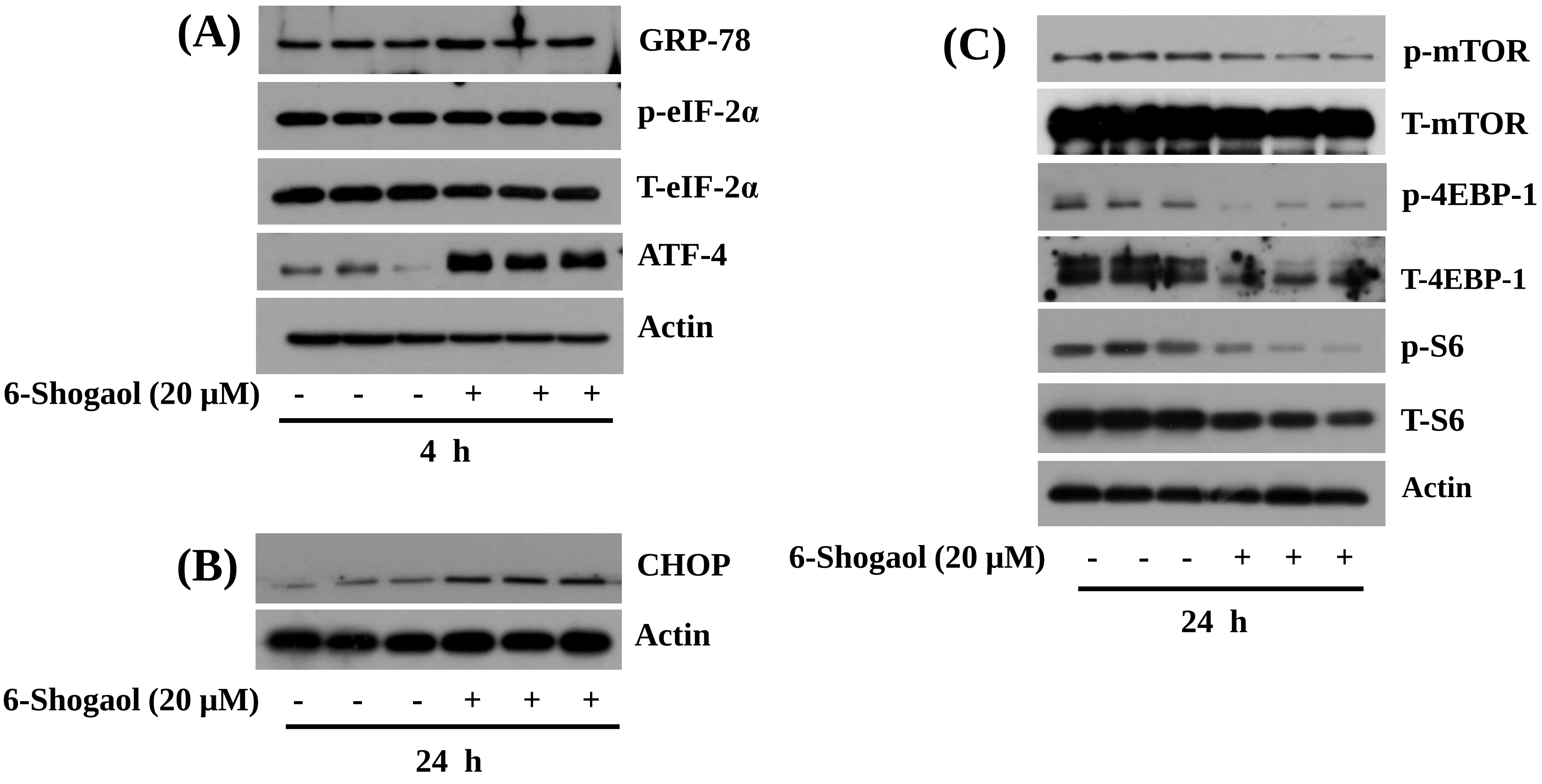

Figure S2. Effects of 6-Shogaol on the expression and phosphorylation levels of GRP-78, eIF-2α, ATF-4, CHOP, mTOR, 4EBP-1, and S6 in SW872 cells. **(A)** SW872 cells were treated with vehicle control (DMSO; 0.1%) or 6-Shogaol (20 μM) for 4 h. Whole cell lysates were prepared and analyzed by Western blotting with respective antibo dies. p-eIF-2α, phosphorylated eIF-2α; T-eIF-2α, total eIF-2α. **(B)** SW872 cells were treated with vehicle control or 6-Shogaol (20 μM) for 24 h. Whole cell lysates were pre pared and analyzed by Western blotting with respective antibodies. **(C)** SW872 cells were treated with vehicle control or 6-Shogaol (20 μM) for 24 h. Whole cell lysates were prepared and analyzed by Western blotting with respective antibodies. p-mTOR, phosphorylated mTOR; T-mTOR, total mTOR; p-4EBP-1, phosphorylated 4EBP-1 ; T-4EBP-1, total 4EBP-1; p-S6, phosphorylated S6; T-S6, total S6.
