# Supplementary material for: Anti-Survival and Pro-Apoptotic Effects of 6-Shogaol on SW872 Human Liposarcoma Cells via Control of the Intrinsic Caspase Pathway, STAT-3, AMPK, and ER Stress
Source: Biomolecules. 2020 Sep 28;10(10):1380. doi: 10.3390/biom10101380 (PMC7650770; doi:10.3390/biom10101380)
Supplement: Supplementary file 1 [file biomolecules-10-01380-s001.zip › biomolecules-936149-supplementary/Table S1.doc]

**Table S1: List of antibodies used for Western blot analysis.**

| **Antibodies** | **Dilution used** | **Source** | **Catalog no.** | |
| --- | --- | --- | --- | --- |
| ***Primary antibodies*** |  |  | |  |
| Procaspase-9 | 1:2,000 | Enzo Life Sciences | | ADI-AAM-139 |
| Procaspase-3 | 1:2,000 | Enzo Life Sciences | | ADI-AAP-113 |
| PARP | 1:2,000 | Roche Diagnostics | | 11835238001 |
| DR-5 | 1:2,000 | Novus Biologicals | | NBP1-45951 |
| p-4EBP-1 (T70) | 1:2,000 | Cell signalling | | #9455 |
| 4EBP-1 | 1:2,000 | Cell signalling | | #9452 |
| p-S6 (S235/236) | 1:4,000 | Cell signalling | | #2211 |
| S6 | 1:4,000 | Cell signalling | | #2317 |
| p-mTOR (S2448) | 1:2,000 | Cell signalling | | #5536 |
| mTOR | 1:2,000 | Cell signalling | | #2972 |
| p-AMPK (T172) | 1:2,000 | Cell signalling | | #2535 |
| AMPK | 1:2,000 | Cell signalling | | #2793 |
| p-LKB-1 (S428) | 1:2,000 | Cell signalling | | #3482 |
| LKB-1 | 1:2,000 | Cell signalling | | #3047 |
| p-eIF-2α (S51) | 1:2,000 | Abcam | | ab32157 |
| eIF-2α | 1:2,000 | Cell signalling | | #9722 |
| p-STAT-3 (Y705) | 1:2,000 | Santa Cruz Biotechnology | | sc-8059 |
| STAT-3 | 1:2,000 | Santa Cruz Biotechnology | | sc-8019 |
| GRP-78 | 1:2,000 | Santa Cruz Biotechnology | | sc-13968 |
| ATF-4 | 1:2,000 | Santa Cruz Biotechnology | | sc-8312 |
| CHOP | 1:2,000 | Santa Cruz Biotechnology | | sc-7351 |
| β-Actin | 1:10,000 | Sigma | | A5441 |
| ***Secondary antibodies*** |  |  | |  |
| Goat anti-rabbit IgG-HRP  Goat anti-mouse-IgG-HRP | 1:5000  1:5000 | Jackson ImmunoResearch  Laboratories  Jackson ImmunoResearch  Laboratories | | 111-035-045  115-035-062 |
